# Socioeconomic disparities in incidence, treatment, and survival of intrahepatic cholangiocarcinoma: insights from a nationwide cohort study in Sweden

**DOI:** 10.1016/j.lanepe.2025.101415

**Published:** 2025-08-05

**Authors:** Juan Vaz, Hannes Hagström, Per Sandström, Malin Sternby Eilard, Magnus Rizell, Ulf Strömberg

**Affiliations:** a School of Public Health and Community Medicine, Institute of Medicine, Sahlgrenska Academy, University of Gothenburg, Sweden; b Department of Medicine, Huddinge, Karolinska Institutet, Stockholm, Sweden; c Department of Medicine, Halland Hospital Halmstad, Halmstad, Sweden; d Unit of Hepatology, Department of Upper GI Diseases, Karolinska University Hospital, Stockholm, Sweden; e Department of Surgery in Linköping and Institution for Biomedical and Clinical Sciences, Linköping University, Linköping, Sweden; f Department of Surgery, Institute of Clinical Sciences, Sahlgrenska Academy, University of Gothenburg, Gothenburg, Sweden; g Region Västra Götaland, Sahlgrenska University Hospital, Department of Transplantation, Gothenburg, Sweden

**Keywords:** Intrahepatic cholangiocarcinoma, Socioeconomic status, Inequalities, Survival

## Abstract

**Background:**

The incidence of intrahepatic cholangiocarcinoma (iCCA) is rising globally, yet the role of socioeconomic status (SES) in shaping disease burden and care within universal healthcare systems remains poorly understood. This study assessed SES-related disparities in the incidence, treatment, and survival of iCCA in Sweden.

**Methods:**

National registry data were used to identify all adult cases of iCCA diagnosed from 2011 to 2021 (n = 1827). Data from the Swedish quality register for liver cancer were cross-linked with socioeconomic and healthcare registers. Household income– categorised as low (lowest national quartile), medium, or high (highest quartile)–was used as the SES indicator. Incidence rates (IRs), treatment patterns, and survival were analysed across income strata.

**Findings:**

The age-standardized IR increased from 1.35 in 2011 to 1.94 per 100,000 person-years in 2021, with the steepest rise observed among men and individuals with low income. Compared to those with high-income, individuals with low income had higher IR ratios of all-stage (1.32, 95% confidence interval [CI]: 1.15–1.52) and late-stage iCCA (1.46, 95% CI: 1.17–1.81). Preventable liver diseases were more prevalent in the low-income patients, while primary sclerosing cholangitis and inflammatory bowel disease were more common among high-income patients. Low income was associated with lower odds of receiving systemic therapy (adjusted odds ratio 0.54, 95% CI: 0.38–0.77) and higher mortality risk among those treated (adjusted hazard ratio 1.34, 95% CI: 1.09–1.65).

**Interpretation:**

Despite universal healthcare access, substantial socioeconomic disparities persist in the incidence, treatment, and outcomes of iCCA in Sweden.

**Funding:**

The 10.13039/501100002794Swedish Cancer Society and The 10.13039/501100001725Royal Swedish Academy of Sciences.

**Research in context**

**Evidence before this study**

A PubMed search was conducted from inception to April 2024 using terms such as “liver cancer”, “intrahepatic cholangiocarcinoma (iCCA)”, “cholangiocarcinoma”, “socioeconomic status”, “socioeconomic position”, “deprivation”, “disparity”, “inequity”, and “inequality.” The majority of relevant studies originated from the United States and consistently reported racial and ethnic disparities in access to iCCA treatment. A few UK-based studies found links between lower socioeconomic status and poorer outcomes among individuals residing in socioeconomically deprived areas. However, large-scale population-based studies focusing specifically on iCCA in high-income countries like Sweden remain scarce.

**Added value of this study**

Using comprehensive national register data–including a dedicated quality register for liver cancer–this study provides new insights into how socioeconomic disparities in care persist despite the presence of a universal healthcare system. The findings underscore inequities in treatment access and management that are not fully addressed by universal coverage alone.

**Implications of all the available evidence**

The existing body of research indicates that universal healthcare can reduce financial barriers but may not be sufficient to eliminate socioeconomic disparities in iCCA treatment and outcomes. Future studies should investigate the underlying mechanisms within healthcare systems that contribute to these inequities and evaluate targeted interventions aimed at promoting more equitable care for patients with iCCA.

## Introduction

Hepatocellular carcinoma (HCC) and intrahepatic cholangiocarcinoma (iCCA) are the most common types of primary liver cancer.^1^ Although the two cancers share certain risk factors, they differ significantly in pathogenesis, clinical presentation, and prognosis.^2^^,^^3^ Over past decades, the global incidence of iCCA has risen,^4^ yet socioeconomic disparities in iCCA incidence, care, and survival outcomes remain largely unexplored among European populations.^5^, ^6^, ^7^

In Sweden, a country with universal healthcare, broad social safety nets, and extensive national registries, studies on HCC have demonstrated that lower socioeconomic status (SES) is associated with higher incidence and worse outcomes.^8^^,^^9^ However, equivalent research on iCCA is lacking. Unlike HCC, iCCA is less closely linked to modifiable risk factors such as viral hepatitis, alcohol-related liver disease (ALD), and metabolic dysfunction-associated steatotic liver disease (MASLD), implying that SES may influence the incidence of iCCA through different mechanisms.^1^

Understanding the relationship between SES and iCCA is crucial to identifying at-risk populations, informing public health interventions, and addressing inequities in cancer care.^5^ This study aims to assess the association between SES and four key aspects of iCCA: incidence, diagnosis, treatment, and survival.

## Methods

### iCCA cases

This study included all patients aged 18 years and older diagnosed with iCCA (International Classification of Diseases, 10th Edition code C22.1) and registered in the Swedish quality register for liver cancer (SweLiv) between 2011 and 2021. SweLiv data were linked to demographic databases and other nationwide registers, such as the National Patient Register (NPR) and the Prescribed Drug Register,^10^ via Sweden’s unique personal identification number. SweLiv, validated against the National Cancer Register in 2014, currently captures over 95% of all cases of liver cancer.^8^

Sociodemographic data were obtained from Statistics Sweden. Each case of iCCA was categorised by calendar year (2011–2021), age-group at diagnosis in 5-year strata (15–19, 20–24, …, 85–89, or 90+), sex (male, female), country of birth (“Nordic” covering Sweden, Norway, Denmark, Finland, Iceland, the Faroe Islands, Greenland, and Åland; and “non-Nordic”), and household income.

Household income was chosen as the main SES indicator, as it provides more complete data than education, especially for immigrants.^11^ Household income, defined as the disposable income per household per consumption unit, was determined for the calendar year preceding iCCA diagnosis and classified into three categories: low, corresponding to the 1st quartile of the national income distribution (“poorest”); medium, including the 2nd and 3rd quartiles; and high, representing the 4th quartile (“wealthiest”).^8^^,^^9^

### Population data for incidence estimation

Population counts for each calendar year were obtained from Statistics Sweden, categorised by sex, 5-year age groups, country of birth, and household income, all of which were defined as described above. These data were used to match denominators to case groups across the defined variables.

### Risk factors and comorbidity

Risk factors diagnosed either prior to or at the time of iCCA diagnosis included biliary conditions (e.g., primary sclerosing cholangitis [PSC], bile duct stones, and cholecystitis); liver diseases (including liver cirrhosis, viral hepatitis B and C, ALD, MASLD, rare liver diseases other than PSC such as autoimmune hepatitis and hemochromatosis); and inflammatory bowel disease (IBD).^1^^,^^2^ Patients could present with multiple risk factors.^2^ Therefore, the prevalences of individual risk factors are reported without treating other plausible causes of iCCA as mutually exclusive, resulting in the sum of counts for underlying risk factors being larger than the total number of patients.

Data on comorbidities were retrieved from the NPR or defined using data from the Prescribed Drug Register. All ICD-codes and other criteria used for the definition of risk factors and comorbidity are presented in the supporting material (Supplementary Table S1). Data on body mass index and tobacco smoke exposure were not available since these variables are neither registered in the NPR nor in SweLiv.

### Diagnostic pathways and waiting times

Diagnostic pathways are recorded in SweLiv at the time of patient registration, based on clinical input. Patients are categorised as diagnosed via surveillance, clinical symptoms, or incidentally, according to a predefined variable completed by the reporting health professional. SweLiv does not provide information on the specific purpose of surveillance (e.g., iCCA detected during HCC surveillance in patients with cirrhosis), the methods used, adherence to surveillance protocols, or the frequency of examinations.

Waiting time that may influence clinical outcomes were obtained from SweLiv. First, the time from initial iCCA suspicion to referral to secondary centre was registered (e.g., interval from primary care referral to hospital, or from one hospital to a specialized surgical team). The time from initial referral to multidisciplinary team cancer conferences (MDTs) was also collected. Finally, the time from the MDT meeting to the first surgical treatment with curative intention or the first visit at an oncology department was measured.

### Performance status, staging and treatment receipt

SweLiv contains data from regional or national MDTs including physicians specialised in biliary tract surgery, oncology, hepatology, radiology, and pathology. Primary diagnosis, staging, risk factors, and treatment recommendations are reported to the SweLiv by healthcare professionals. Primary diagnosis and staging status are updated if needed after examination of surgical resected tissue, or upon additional clinical data available after MDTs.

Eastern Cooperative Oncology Group performance status (ECOG PS) was retrieved from SweLiv. Staging at diagnosis was determined using the 8th edition of the Union for International Cancer Control Tumour-Node-Metastasis (TNM) classification for iCCA.^12^ TNM data were retrieved from SweLiv, or from the National Cancer Register and corresponding to the staging before surgical procedures.

Early-stage was defined as either carcinoma in situ or a single primary tumour (T1a or T1b) without vascular invasion and no evidence of regional node or distant metastasis (N0 and M0). Late-stage was defined as distant metastasis (M1), independent of primary tumour or regional node stage, while all remaining cases (T2-T4 stages with N0 or N1 but M0) were classified as intermediate-stage. A more detailed description of the 8th Edition of TNM for iCCA is provided in the supporting material (Supplementary Table S1). Data on patterns of growth and differentiation grades were not available.

Received treatments were categorised into four groups: surgical, systemic therapy, other palliative treatment, and best supportive care (BSC). Surgical procedures included liver resection and liver transplantation, but excluded exploratory procedures, and were identified through SweLiv. In Sweden, surgical treatments for iCCA are considered to have curative intent by clinical practice and national guidelines. These procedures were further validated by cross-linking to the NPR through specific procedural codes.^10^ Data on resection margins (e.g., R0, R1, R2) were not available for most patients and were therefore not included in survival analyses described below.

Systemic therapy was defined in patients who received chemotherapy or immunotherapy without undergoing surgery, either before or after. Data on chemotherapy and immunotherapy regimens were first introduced to SweLiv in 2020. Therefore, to improve case identification, systemic therapy was also defined based on patients having four or more visits to an oncology department following the date of iCCA diagnosis, where either a procedural code for chemotherapy or immunotherapy was recorded, or the timing of visits corresponded to known cisplatin-gemcitabine protocols (day 1, day 8 and new cycle in day 22).^13^ To validate this approach, the definition was compared with registry-recorded systemic therapy data for patients diagnosed between 2020 and 2021, showing a concordance of 95%, supporting the robustness of the classification.

Other palliative treatments included locoregional treatments, such as radiotherapy and were defined in patients who received neither surgical nor systemic therapy. Patients with no antitumour treatment recorded were classified as receiving BSC.

### Statistical analysis

Age-standardised incidence rates (ASIRs) were calculated using the Revised European Standard Population from 2013 (ESP-2013).^14^ The original ESP-2013 weights were truncated to exclude age groups <15 years. The remaining weights for ages 15–19 through 90+ were proportionally rescaled to sum 100,000. This approach ensures comparability while avoiding bias from non-included age-strata. Using Byar’s method,^15^ we calculated a 95% confidence interval (CI) around each ASIR. Age-specific incidence rates (IRs) were also calculated. Both ASIRs and age-specific IRs were presented using Cartesian coordinate planes: ASIRs per 100,000 person-years vs. year of diagnosis, and age-specific IRs per 100,000 person-years vs. age-group. For ASIRs, restricted cubic splines were used to fit lines to graphically illustrate time trends during the study period.

Poisson regression, with population data denominators log-transformed and used as offsets, was employed to assess the relationships between sociodemographic factors in the adult population of Sweden and IRs of all-stage, early-stage, and intermediate- or late-stage of iCCA. Incidence rate ratios (IRRs) with 95% CIs were thereby estimated –for group comparisons. Independent (group) variables included sex, country of birth and household income, adjusted for age-group and calendar year at diagnosis. To assess the robustness of the choice of model, corresponding negative binomial regression analyses—accounting for potential overdispersion in the count data—were performed. The estimates from negative binomial models were virtually identical to those from Poisson regression (results not shown).

Univariable and multivariable logistic regression models were used to estimate odds ratios (ORs) and adjusted odds ratios (aORs) for: i) receiving surgical treatment, ii) receiving systemic therapy, and iii) receiving other palliative treatments among patients diagnosed with iCCA. Multivariable models were adjusted for age, sex, country of birth, household income, liver cirrhosis, TNM stage, and ECOG PS.

Each patient was followed until the date of death, emigration from Sweden, or May 23, 2024, whichever occurred first. Kaplan–Meier estimates with Greenwood 95% CIs were used to determine median survival and survival probabilities across sociodemographic, TNM-stage, and treatment groups. Cox regression models provided adjusted hazard ratios (aHRs) for all-cause mortality.

The final Cox model was initially planned to adjust for age, sex, country of birth, household income, and treatment (surgery, systemic therapy, other palliative treatments, or BSC). However, testing the proportional hazards assumption using Schoenfeld residuals with *estat phtest* revealed a violation for the treatment variable. Consequently, the final Cox model was stratified by treatment option, with the remaining covariates adjusted as described above.

Sweden’s Cancer Patient Pathway for iCCA was introduced in 2016. To assess potential changes in iCCA outcomes over time (all causes), the study period was divided into two groups (2011–2015, 2016–2021). Using logistic and Cox regression models as described above, treatment options and mortality risk were evaluated, with “period of diagnosis” included as a categorical variable and 2011–2015 as the reference.

### Handling of missing data

Missing data primarily concerned ECOG PS, which was uncertain in 210 patients (13%). Of these, only 94 patients (5% of the total cohort) received surgical treatment (n = 11), systemic therapy (n = 56), or other palliative treatment (n = 27). Given that surgical, systemic, and other palliative therapies are typically reserved to patients with ECOG PS 0–1, ECOG 0 was assigned to patients undergoing surgery without comorbidities, and ECOG 1 to the remaining patients in this group. For the 116 patients with uncertain ECOG who received only BSC an ECOG ≥2 was assigned. As missingness for other variables was minimal (<3% for TNM stage), a complete case analysis was applied in the multivariable models.

All tests were conducted using Stata v.19 (StataCorp, College Station, TX, USA).

### Ethics approval

This study was approved by the Central Ethical Review Board in Sweden (decision number 2023-04555-01 and 2024-05174-02). Due to its register-based nature, patient consent was not required.

### Role of the funding source

The funders had no role in study design, data collection, data analysis, data interpretation, preparation of the report, or decision to publish.

## Results

### Patient characteristics

A total of 1827 patients with iCCA were identified, with a median age of 70 years (IQR: 62–76; range: 18–95), and 584 patients (32%) were younger than 65 years at diagnosis. Half of the cohort (919; 50.3%) were male, and most (90%) were born in Nordic countries. Regarding household income, 47% had a medium level, while 30% and 23% had low and high incomes, respectively (Table 1). Compared to those with high income, patients with low income were more likely to be older (median age 74 vs. 66 years, p < 0.001), female (57% vs. 44%, p < 0.001), and born outside the Nordic region (16% vs. 5%, p < 0.001).

**Table 1:** Baseline characteristics of 1827 patients diagnosed with intrahepatic cholangiocarcinoma in Sweden between 2011 and 2021.

	Household income level			
	Low	Medium	High	Total
	538 (30)	865 (47)	424 (23)	1827 (100)
**Male sex**	229 (43)	452 (52)	238 (56)	919 (50)
**Median age**	74 (67–80)	70 (62–76)	66 (59–72)	70 (62–76)
**Age group**				
18–64	115 (21)	275 (32)	194 (46)	584 (32)
65–79	278 (52)	469 (54)	206 (49)	953 (52)
80+	145 (27)	121 (14)	24 (5)	290 (16)
**Country of birth**				
Nordic	452 (84)	792 (92)	401 (95)	1645 (90)
Non-Nordic	86 (16)	73 (8)	23 (5)	182 (10)
**Risk factors**				
PSC	11 (2)	62 (7)	39 (9)	112 (6)
Rare liver diseases	12 (2)	21 (2)	10 (2)	43 (2)
Hepatitis B	17 (3)	18 (2)	2 (<1)	37 (2)
Hepatitis C	37 (7)	33 (4)	11 (3)	81 (4)
IBD	21 (4)	69 (8)	52 (12)	142 (8)
PSC + IBD	10 (2)	51 (6)	29 (7)	90 (5)
Liver cirrhosis	89 (17)	94 (11)	56 (13)	239 (13)
ALD	67 (12)	62 (7)	31 (7)	160 (9)
Bile duct stones/cholecystitis	82 (15)	104 (12)	56 (13)	242 (13)
MASLD	104 (19)	137 (16)	52 (12)	293 (16)
No identified risk factors	260 (48)	488 (56)	251 (59)	999 (55)
**Diagnostic pathways**				
Surveillance	38 (7)	56 (7)	32 (8)	126 (7)
Clinical symptoms	390 (73)	643 (74)	310 (73)	1343 (74)
Incidental by radiology	82 (15)	136 (16)	59 (14)	277 (15)
Unknown	25 (5)	30 (3)	23 (5)	81 (4)
**Waiting time (in days)**				
First suspicion to referral	4 (0–16)	4 (0–19)	4 (0–17)	4 (0–17)
Referral to MDT	9 (5–18)	9 (6–18)	8 (6–16)	9 (6–18)
MDT to surgery	41 (26–63)	33 (20–60)	39 (21–55)	34 (21–60)
MDT to oncology clinic	27 (18–48)	28 (14–55)	22 (11–55)	27 (14–54)
**Primary tumour (T)**				
Median size (mm)	69 (37–100)	60 (38–100)	60 (35–93)	62 (36–100)
TX	20 (4)	33 (4)	19 (5)	72 (4)
T1	155 (29)	256 (29)	115 (27)	526 (29)
T2	181 (34)	330 (38)	169 (40)	680 (37)
T3	72 (13)	119 (14)	56 (13)	247 (14)
T4	110 (20)	127 (15)	65 (15)	302 (16)
**Regional lymph node (N)**				
NX	43 (8)	56 (6)	21 (5)	120 (6)
N0	310 (58)	510 (59)	251 (59)	1071 (59)
N1	185 (34)	299 (35)	152 (36)	636 (35)
**Distant metastases (M)**				
M0	311 (58)	530 (61)	257 (61)	1098 (60)
M1	227 (42)	335 (39)	167 (39)	729 (40)
**Staging (TNM 8th)**				
Early, stage I	102 (19)	183 (21)	83 (20)	368 (20)
Intermediate, stages II–III	193 (36)	326 (38)	161 (38)	680 (37)
Late, stage IV	227 (42)	335 (39)	167 (39)	729 (40)
Cannot be assessed	16 (3)	21 (2)	13 (3)	50 (3)
**ECOG PS**				
0	95 (18)	259 (30)	156 (37)	510 (28)
1	124 (23)	219 (25)	97 (23)	440 (24)
2	212 (39)	221 (26)	92 (22)	525 (29)
>2 or uncertain	107 (20)	199 (19)	79 (18)	352 (19)
**Comorbidities**				
Arterial hypertension	219 (41)	282 (33)	145 (34)	646 (35)
Type 2 diabetes	169 (31)	211 (24)	85 (20)	465 (25)
Hyperlipidaemia	224 (42)	372 (43)	149 (35)	745 (41)
Coronary artery disease	88 (16)	102 (12)	41 (10)	231 (13)
COPD	69 (13)	60 (7)	12 (3)	141 (8)
Cancer other than iCCA^a^	43 (8)	75 (9)	41 (10)	159 (9)
**Treatment**				
Surgical	86 (16)	192 (22)	102 (24)	380 (21)
Systemic therapy	107 (20)	260 (30)	136 (32)	503 (28)
Other palliative treatments	49 (9)	108 (13)	52 (12)	209 (11)
Best supportive care	296 (55)	305 (35)	134 (32)	735 (40)

ALD: alcohol-related liver disease; COPD: chronic obstructive pulmonary disease; ECOG PS: Eastern Cooperative Oncology Group performance status; iCCA: intrahepatic cholangiocarcinoma; MASLD: metabolic dysfunction-associated steatotic liver disease; IBD: inflammatory bowel disease; PSC: primary sclerosing cholangitis.

a Diagnosed within the two years prior to the date of iCCA diagnosis.

While TNM stage at diagnosis was similar across income groups, patients with high income were more often classified as ECOG PS 0 compared to those with low income (37% vs. 18%, p < 0.001). Patients in the low-income group had higher prevalences of viral hepatitis, ALD, MASLD, and liver cirrhosis. In contrast, patients with high income −46% of whom were younger than 65 years– more frequently had PSC and IBD, either alone or in combination. Overall, 55% of patients had no identifiable aetiological risk factors for iCCA. When stratified by income level, patients without identifiable aetiological risk had similar demographic and clinical characteristics compared to the overall cohort (Supplementary Table S2).

### Incidence

**Fig. 1:**
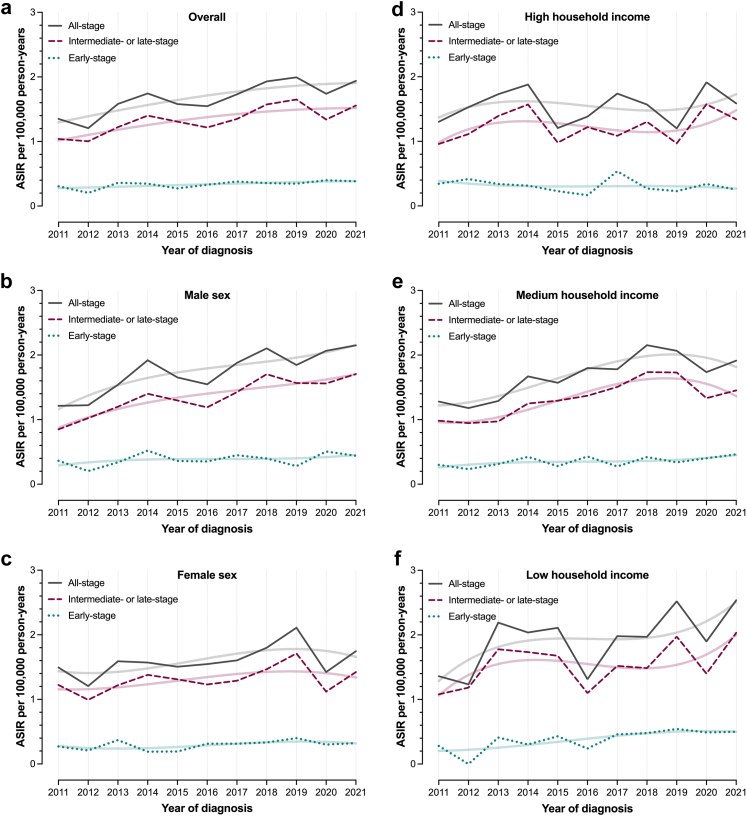
Age-standardised incidence rates (ASIRs) of intrahepatic cholangiocarcinoma (iCCA) in Sweden between 2011 and 2021. Panel (a) presents ASIRs of all-stage, early-stage and intermediate- or late-stage iCCA across the study period, while panels (b) and (c) present ASIRs for men and women, respectively. Panels (d–f) present ASIRs for individuals with high, medium, and low household income, respectively. Restricted cubic splines were used to fit lines representing changes in the all-stage and stage-specific ASIRs of iCCA during the study period for each individual panel (a–f).

**Fig. 2:**
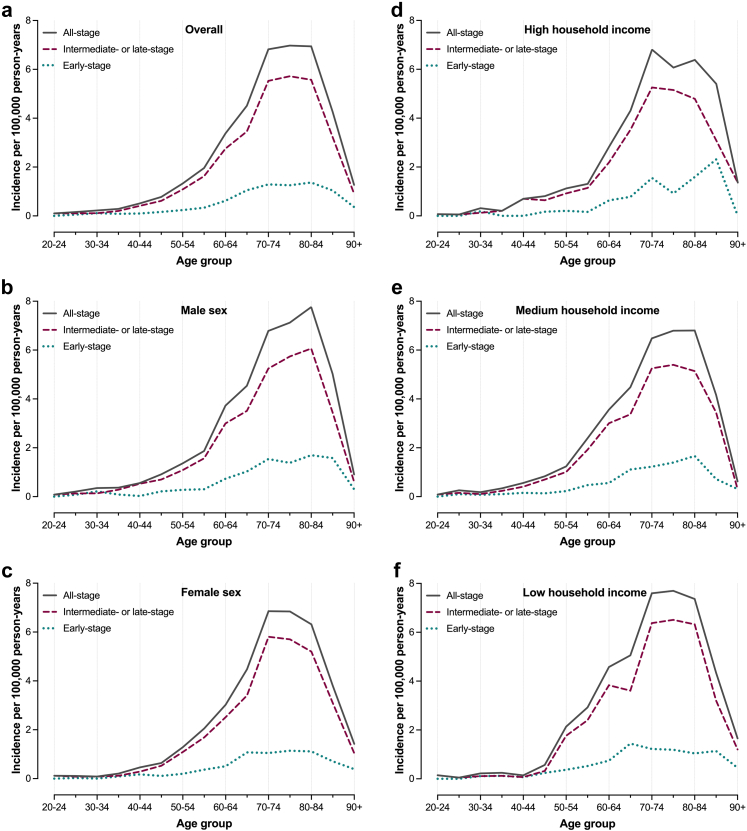
Age-specific incidence rates (IRs) of intrahepatic cholangiocarcinoma (iCCA) in Sweden between 2011 and 2021. Panel (a) presents IRs of all-stage, early-stage and intermediate- or late-stage iCCA across the study period, while panels (b) and (c) present IRs for men and women, respectively. Panels (d–f) present IRs for individuals with high, medium, and low household income, respectively.

The ASIR of all-stage iCCA during the study period was 1.68 per 100,000 person-years (95% CI: 1.61–1.76); 1.76 (95% CI: 1.65–1.88) for men and 1.61 (95% CI: 1.50–1.71) for women. The ASIR increased from 1.35 (95% CI: 1.11–1.59) in 2011 to 1.94 (95% CI: 1.68–2.20) in 2021. Fig. 1, Fig. 2 present ASIRs and age-specific IRs of all-stage and stage-specific iCCA for the total population, as well as stratified by sex and household income group. ASIRs displayed somewhat varying time-trends by stage at diagnosis and across income groups. Men and individuals with low household income showed a trend towards increasing ASIRs of all-stage iCCA, driven by parallel increments in ASIRs of early- and intermediate- or late-stage iCCA.

A total of 149 patients (8%) were diagnosed with iCCA before the age of 50. The age-specific incidence rate for patients <50 years was 0.30 per 100,000 person-years (95% CI: 0.26–0.36), and did not change significantly over the study period. No statistically significant income-related differences were observed within this age subgroup.

**Fig. 3:**
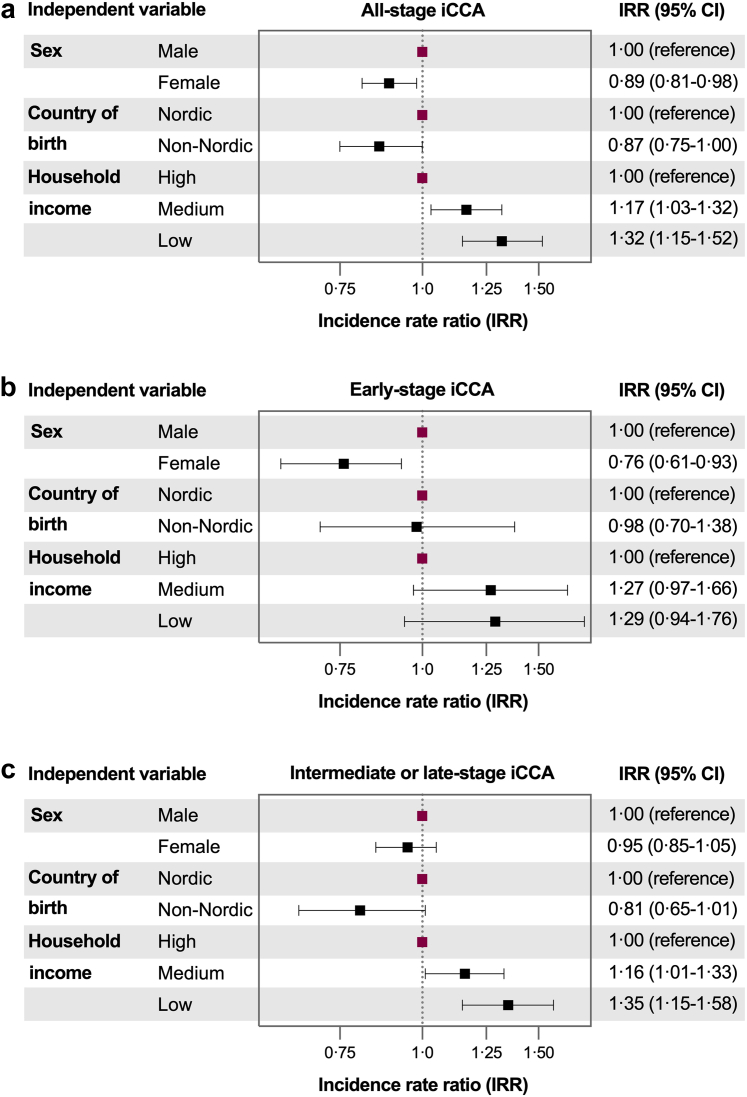
Associations of the individual level variables sex, country of birth, and household income with incidence rates of all-stage (panel a), early-stage (panel b) and intermediate- or late-stage (panel c) of intrahepatic cholangiocarcinoma (iCCA) among adults (18 years and older) in Sweden between 2011 and 2021. The fully adjusted models included adjustments for age and calendar year, as well as for all included independent variables.

Fig. 3 presents adjusted IRR estimates from multivariable models, adjusted for age and calendar year. Household income was significantly associated with iCCA IRs. Compared to individuals with high household income, those with low income had higher IRRs for all-stage iCCA (1.32, 95% CI: 1.15–1.52) and intermediate- or late-stage iCCA (1.35, 95% CI: 1.16–1.57), independent of other covariates. When examining intermediate- and late-stage IRs separately, the higher IRR of all-stage iCCA among individuals with low income was primarily driven by a notably higher IRR for late-stage (1.46, 95% CI: 1.17–1.81) in this group.

### Treatment receipt

After initial MDT evaluation, 514 patients (28%) were recommended surgical treatment, with higher proportions among those with high or medium income compared to those with low income (32% and 30% vs. 22%, p < 0.001). Of these, 380 (74% of those recommended, 21% of the whole cohort) underwent surgery, including 369 resections and 11 transplantations. The proportion of patients not receiving the recommended surgery did not differ significantly by income group (data not shown).

Systemic therapy was recommended for 615 patients (34%), again more frequently among those with high or medium income than among those with low income (38% and 36% vs. 27%, p < 0.001). Of these patients, 503 (82% of those recommended, 28% of the whole cohort) received systemic therapy. The proportion of patients not receiving the recommended systemic therapy was higher in the low-income group compared to those with medium or high income (26% vs. 15% and 16%, p = 0.016).

Overall, patients with high income were more often treated with surgery compared to those with low income (24% vs. 16%, p = 0.002). Similarly, patients with high income received more often systemic therapy compared to those with low income (32% vs. 20%, p < 0.001) (Table 1).

ORs and aORs for receiving surgical, systemic therapy, or other palliative treatments are presented in the supporting information (Supplementary Tables S3–S5). Low household income, compared to high income, was associated with a lower likelihood of receiving systemic therapy (aOR 0.54, 95% CI: 0.38–0.77). No sociodemographic variable was associated with the likelihood of receiving surgery or other palliative treatments.

### Survival

During a total follow-up of 2780 person-years, 1655 patients (91%) died. Median survival time was 8.6 months (95% CI: 7.7–9.2), and the 1-, 2-, and 5-year survival probabilities were 0.41 (95% CI: 0.39–0.43), 0.23 (95% CI: 0.21–0.25), and 0.10 (95% CI: 0.09–0.12), respectively (Table 2). Patients with low household income had slightly shorter median survival and lower 1-year survival probability compared to those with high income. Survival outcomes for each sociodemographic group, stratified by treatment type, are presented in Supplementary Table S6. Among patients who received surgery, women had longer median survival and higher 5-year survival probabilities. Patients with low income in this group showed somewhat better survival than those with medium or high income, although the differences were not statistically significant.

**Table 2:** Survival probabilities of patients diagnosed with intrahepatic cholangiocarcinoma in Sweden between 2011 and 2021.

	N	Deaths	Survival probability (95% CI)			Median survival in months (95% CI)
			1-year	2-year	5-year	
**Overall**	1827	1655	0.41 (0.39–0.43)	0.23 (0.21–0.25)	0.10 (0.09–0.12)	8.6 (7.7–9.2)
**Sex**						
Male	919	840	0.40 (0.37–0.43)	0.22 (0.19–0.25)	0.09 (0.07–0.11)	8.4 (7.4–9.2)
Female	908	815	0.42 (0.39–0.46)	0.24 (0.21–0.27)	0.12 (0.10–0.14)	8.6 (7.4–9.9)
**Country of birth**						
Nordic	1645	1495	0.41 (0.39–0.44)	0.22 (0.20–0.24)	0.10 (0.09–0.12)	8.4 (7.6–9.2)
Non-Nordic	182	160	0.42 (0.34–0.49)	0.29 (0.24–0.35)	0.11 (0.07–0.16)	9.2 (7.8–12)
**Household income**						
High	424	380	0.47 (0.43–0.52)	0.26 (0.22–0.30)	0.13 (0.10–0.16)	10 (8.4–13)
Medium	865	786	0.43 (0.39–0.46)	0.23 (0.20–0.26)	0.10 (0.08–0.12)	8.9 (8.1–10)
Low	538	489	0.34 (0.30–0.38)	0.20 (0.17–0.24)	0.09 (0.06–0.12)	6.3 (5.3–7.8)
**TNM-stage**^a^						
I	368	276	0.73 (0.68–0.77)	0.52 (0.47–0.57)	0.28 (0.23–0.33)	25 (22–30)
II	293	246	0.56 (0.50–0.61)	0.36 (0.30–0.41)	0.19 (0.15–0.24)	15 (12–18)
III	387	359	0.42 (0.37–0.47)	0.19 (0.15–0.23)	0.07 (0.05–0.10)	9.3 (7.4–11)
IV	729	725	0.20 (0.17–0.23)	0.06 (0.04–0.08)	<0.01 (−)	4.3 (3.8–4.8)
**Treatment**						
Surgery	380	242	0.86 (0.82–0.89)	0.66 (0.60–0.70)	0.42 (0.37–0.47)	43 (35–50)
Systemic therapy	503	483	0.58 (0.54–0.62)	0.24 (0.20–0.28)	0.04 (0.03–0.06)	14 (13–15)
Other Palliative	209	208	0.12 (0.09–0.15)	0.04 (0.02–0.06)	0.01 (0.01–0.02)	3.8 (3.4-4.3)
BSC	735	722	0.16 (0.13–0.19)	0.07 (0.05–0.08)	0.02 (0.01–0.03)	2.9 (2.7–3.5)

BSC: best supportive care; CI: confidence interval.

a TNM-stage could not be defined for 50 patients (3% of total cohort).

**Fig. 4:**
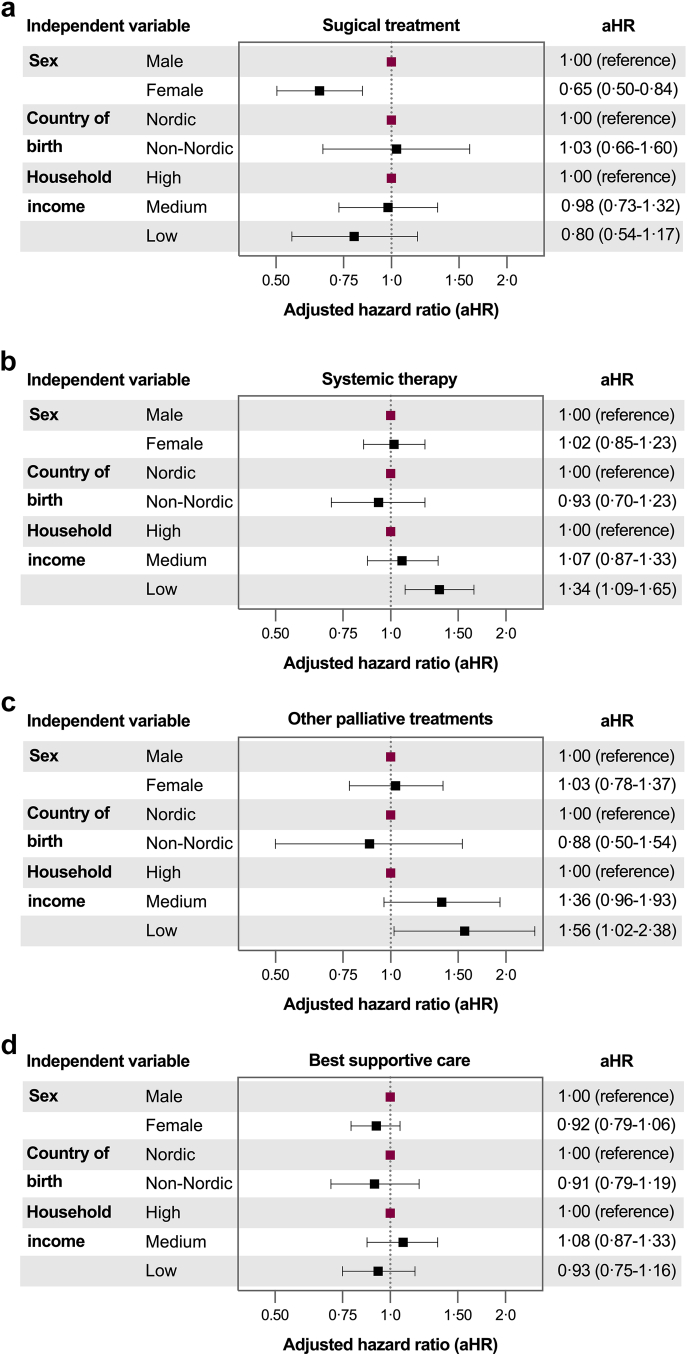
Results from multivariable Cox regression models showing adjusted hazard ratios for overall mortality in patients with intrahepatic cholangiocarcinoma in Sweden from 2011 to 2021. All models were adjusted for age at diagnosis. Panel (a) presents results for patients treated with surgery, while panels (b), (c), and (d) present results from patients treated with systemic therapy, other palliative treatments, and best supportive care, respectively.

In the fully adjusted Cox models, low household income was associated with an increased mortality risk among patients receiving systemic therapy (aHR 1.34, 95% CI: 1.09–1.65) or other palliative treatments (aHR 1.56, 95% CI: 1.02-2.38) (Fig. 4 and Supplementary Table S7). No significant associations were found between age or country of birth and mortality risk. Among patients receiving surgery, women had lower mortality risk compared to men (aHR 0.65, 95% CI: 0.50–0.84).

Multivariable models showed no association between the period of diagnosis and the likelihood of receipt of surgical treatment, systemic therapy, or other palliative treatments. Similarly, median survival and 1-, 2-, and 5-year survival probabilities improved only marginally between 2011-2015 and 2016–2021, with differences falling within the margin of statistical error (data not shown).

## Discussion

This Swedish nationwide study reveals clear associations between SES and the incidence of iCCA, with individuals in the lowest income group exhibiting the highest risk. In line with global trends, this study shows an increasing incidence of iCCA over the study period, with most cases diagnoses at intermediate or late stages.^2^^,^^16^ Notably, the increase in incidence was most pronounced among men and individuals with low SES. While SES was not associated with receipt of surgical treatment, patients with low household income were less likely to receive systemic therapy and had worse survival outcomes within this treatment group and in patients receiving other palliative treatments. Importantly, findings of this study also shown that none of the measured outcomes –treatment receipt and survival– have improved in Sweden during the study period.

Studies from other European nations reveal similar links between lower SES and higher liver cancer incidence,^5^^,^^17^ although specific data on iCCA remain limited. SES may indirectly influence iCCA incidence through comorbid conditions and lifestyle factors. Obesity, metabolic diseases, and environmental exposures –potentially more prevalent in low-income groups– could interact with genetic predispositions to increase risk for iCCA.^2^^,^^18^ Other established risk factors, such as liver cirrhosis and chronic viral hepatitis, are also more prevalent in individuals with low SES.^19^ In our study, patients with low SES more frequently had MASLD, ALD, liver cirrhosis, and viral hepatitis compared to those with high SES. However, all these differences were modest, and most patients, regardless of SES group, lacked identified risk factors for iCCA.

Research from the UK highlights SES-related disparities in iCCA outcomes, including delayed diagnosis and reduced access to treatment, particularly in socioeconomically deprived neighbourhoods.^6^^,^^7^^,^^20^, ^21^, ^22^, ^23^ Patients from lower SES groups may also have limited symptom awareness, and difficulties navigating the healthcare system.^22^^,^^23^ These challenges are particularly relevant in liver disease, which are often asymptomatic until late stages and carry stigma.^19^ However, in this study, no major differences were observed in the documented diagnostic pathways across SES groups, likely reflecting the high prevalence of patients without known risk factors in all SES groups.

Although most iCCA cases occurred in older adults, nearly one in twelve patients were diagnosed before age 50. These early-onset cases were more common among high-income individuals, likely reflecting the broader age-income gradient observed in the cohort. Age-specific incidence in those under 50 remained stable over time, and income-related differences in this subgroup were not statistically significant –likely due to the rarity of early-onset disease and limited statistical power. Nonetheless, the presence of iCCA in younger patients underscores the need for timely diagnostic workup, particularly among individuals with risk factors such as PSC.

Overall, high-income patients were younger at diagnosis, which may be explained by the higher prevalence of PSC and IBD, conditions more common in younger populations and more often detected through surveillance. Similar patterns were observed even among patients without identifiable risk factors, suggesting that SES may influence healthcare-seeking behaviour, with earlier presentation and diagnosis more common in higher-income groups. Comorbidity burden also differed by SES; patients with higher income had lower rates of cardiometabolic disease and COPD, potentially contributing to better overall health and more favourable ECOG PS at diagnosis, despite similar TNM staging across income groups.

Although patients with higher income were more likely to undergo surgery in unadjusted analyses, this association was not statistically significant after adjusting for clinical variables. This likely reflects the dominant role of tumour stage and ECOG performance status in determining surgical eligibility. Since lower-income patients more often presented with poorer performance status, they may have been less likely to be considered operable. Moreover, resection margin status (R0, R1, R2) was not available in the registry, limiting the assessment of surgical completeness and its potential influence on outcomes. These unmeasured factors may help explain the absence of socioeconomic differences in adjusted survival among patients who received surgery.

In contrast, patients with low income were significantly less likely to receive systemic therapy, even after adjustment for ECOG and TNM stage. Notably, 18% of patients recommended for systemic therapy did not receive treatment, with a higher proportion of non-treatment observed in the low-income group. Potential explanations include a higher burden of unmeasured comorbidities, more aggressive tumour biology with rapid progression, or social and logistical barriers such as travel limitations, difficulty adhering to treatment schedules, or attitudinal factors among patients or providers that influence treatment initiation.

These findings, observed within the context of Sweden’s universal, tax-funded healthcare system, highlight that socioeconomic disparities in cancer care can persist even in systems designed to promote equity.^24^ Sweden offers comprehensive coverage with minimal out-of-pocket costs, yet differences in disease presentation and treatment receipt by income level were still observed. Similar challenges have been reported in other high-income countries with universal healthcare.^5^ In contrast, countries lacking universal healthcare –such as the United States– typically face even more pronounced disparities, often driven by insurance status, financial barriers, and fragmented care delivery.^25^ Our findings underscore the need for equity-focused policies not only to ensure access but also to address upstream determinants of health, including health literacy, navigation support, and structural barriers to early diagnosis and treatment.^5^^,^^19^

The persistent challenges in the diagnosis and treatment of biliary tract cancer (BTC), including late-stage detection, limited treatment options, and significant variation in access to care across countries in Europe have being recently highlighted.^4^ These issues are particularly relevant in the Swedish setting, where socioeconomic differences in stage at diagnosis and receipt of treatment were found in this study. Furthermore, access to novel therapies in Sweden may be uneven. As shown in a recent comparative analysis,^26^ Sweden lags behind several other European countries in the implementation of newly approved treatments for BTC, potentially due to delays in national reimbursement processes or regional differences in clinical practice. Given the high costs associated with immunotherapies and next-generation sequencing (NGS),^27^^,^^28^ it is imperative that patients with low SES are afforded equal access to these emerging treatment options.

To address the socioeconomic disparities identified in this study, equity must be embedded not only in healthcare financing but also in the design and delivery of cancer care pathways. Within universal systems like Sweden’s, reducing inequalities will require a multi-level approach.^5^^,^^19^ At the patient level, efforts should focus on improving liver disease awareness, reducing stigma, and supporting early help-seeking behaviour through health literacy campaigns and primary care engagement.^5^^,^^19^ At the system level, structured referral protocols and patient navigation programs may reduce barriers to timely diagnosis and treatment, particularly for patients with low income or complex care needs.^4^, ^5^, ^6^^,^^19^

Access to NGS and advanced treatments is often concentrated in tertiary centres and may be limited by geographic location, referral patterns, or differences in clinician and patient awareness. Patients from lower socioeconomic backgrounds may be less likely to be referred for molecular testing, less able to participate in clinical trials, or face challenges in navigating reimbursement for high-cost treatments. Without proactive policy measures, these factors risk reinforcing inequities in who benefits from advances in cancer care.

To mitigate this risk, system-level efforts are needed to ensure equitable access to molecular diagnostics and personalised treatments. These include national reimbursement pathways for NGS, integration of testing into standard diagnostic workflows, expansion of referral networks for patients in underserved areas, and transparency in clinical decision-making criteria. Routine equity audits of access to precision oncology and public reporting of disparities in molecular testing and treatment uptake could also support more inclusive implementation. As the therapeutic landscape for iCCA evolves,^26^^,^^29^ ensuring that innovations reach all patients –regardless of socioeconomic background– may be critical to improving population-level outcomes and closing equity gaps. Addressing disparities in the next generation of cancer care will demand deliberate, equity-oriented policies that go beyond coverage and actively dismantle structural and informational barriers to high-quality care.

The strength of this study lies in its comprehensive nationwide coverage, encompassing over 95% of all iCCA cases diagnosed in Sweden during a contemporary, extended study period. The risk of selection bias is minimal due to the country’s universal access to healthcare services. By linking SweLiv data with several national registers, the dataset was enriched with detailed clinical and sociodemographic information. This integrated approach enhances the scope and depth of the findings beyond what is typically feasible in register-based studies.

Several features further support the reliability of the results: (a) no missing data on key sociodemographic variables and less than 3% missing on staging; (b) use of clearly defined variables and robust statistical methods, yielding consistent results; (c) data collection from MDTs, minimising the risk of misclassification between iCCA, perihilar cholangiocarcinoma, and HCC; and (d) the large sample size and long observation period, which enabled precise estimates of iCCA incidence across sociodemographic strata.

However, some limitations should be acknowledged. First, country of birth could not be disaggregated by nationality or ethnicity, limiting more granular assessment of population subgroups. Second, no formal sensitivity analyses were conducted using alternative definitions of SES. Nevertheless, household income was defined using national quartiles, a method that allows for comparability across time and demographic groups and has being used successfully in prior population-based studies in Sweden.^8^^,^^9^ Third, tumour resectability and operability were not captured in the registry; TNM stage was used to evaluate severity, limiting precision in assessing whether disparities were driven by delay diagnosis, differences in tumour biology, or comorbidities affecting treatment eligibility. Forth, data on specific systemic therapies were unavailable before 2020, which may have led to misclassification between “systemic treatment” and “other palliative treatments” and between “other palliative treatments” and BSC. To address this, a registry-based algorithm was used. This algorithm showed >95% concordance with registry-defined systemic therapy in the 2020–2021 subset, supporting its interval validity. Nevertheless, the absence of detailed treatment data in earlier years underscores the need for improved capture of systemic therapies in national cancer registries to enable more granular analyses of treatment equity and outcomes. Finally, although clear associations between SES and iCCA incidence, treatment and outcomes were observed, the registry data did not allow investigation into the more specific underlying mechanisms –such as health literacy, care-seeking behaviour, or cultural factors– that may mediate these disparities.

This study highlights persistent socioeconomic disparities in the incidence and management of iCCA in Sweden. Individuals with low household income had higher incidences of all-stage and late-stage disease, were less likely to receive systemic therapy, and experienced poorer survival –despite universal healthcare access. These findings underscore the urgent need to address structural and clinical barriers to equitable cancer care and to ensure that all patients, regardless of socioeconomic background, have timely access to diagnostics and emerging therapies in the evolving era of precision oncology.

## Contributors

The work reported in the article has been performed by the authors, unless clearly specified in the text. Specific author contributions: JV: Conceptualization, Methodology, Formal analysis, Investigation, Data acquisition and curation, Visualization, Writing—Original Draft. HH: Conceptualization, Writing-Original Draft, Supervision. PS, MR and MSE: Conceptualization, Writing-review and editing. US: Conceptualization, Methodology, Data acquisition, Writing—Original Draft, Funding acquisition, Main supervision. JV and US have direct access to the data. All authors have contributed and approved the final version.

## Data sharing statement

Datasets generated and analysed during the current study are not publicly available due to legal restrictions, but additional analyses may be requested to the corresponding author upon reasonable request.

## Declaration of interests

JV has received consulting fees from Roche and Astra Zeneca and a research grant from Eisai. HH:s institutions have received research funding from Astra Zeneca, EchoSens, Gilead, Intercept, MSD, Novo Nordisk, Takeda and Pfizer. He has served as consultant, speaker or on advisory boards for Astra Zeneca, Boehringer Ingelheim, Bristol Myers-Squibb, GSK, Echosens, Ipsen, MSD and Novo Nordisk and has been part of hepatic events adjudication committees for Arrowhead, Boehringer Ingelheim, KOWA and GW Pharma. AuthorMSE has received consulting fees from Baxter, Astra Zeneca and Eisai. PS, MR and US have no conflicts of interest.

## References

[1] European Association for the Study of the Liver (2023). EASL-ILCA clinical practice guidelines on the management of intrahepatic cholangiocarcinoma. J Hepatol.

[2] Banales J.M., Marin J.J.G., Lamarca A. (2020). Cholangiocarcinoma 2020: the next horizon in mechanisms and management. Nat Rev Gastroenterol Hepatol.

[3] Llovet J.M., Kelley R.K., Villanueva A. (2021). Hepatocellular carcinoma. Nat Rev Dis Primers.

[4] Rimassa L., Khan S., Groot Koerkamp B. (2025). Mapping the landscape of biliary tract cancer in Europe: challenges and controversies. Lancet Reg Health Eur.

[5] Kondili L.A., Lazarus J.V., Jepsen P. (2024). Inequities in primary liver cancer in Europe: the state of play. J Hepatol.

[6] Liao W., Coupland C.A.C., Innes H. (2023). Disparities in care and outcomes for primary liver cancer in England during 2008-2018: a cohort study of 8.52 million primary care population using the QResearch database. eClinicalMedicine.

[7] Tataru D., Khan S.A., Hill R. (2024). Cholangiocarcinoma across England: temporal changes in incidence, survival and routes to diagnosis by region and level of socioeconomic deprivation. JHEP Rep.

[8] Vaz J., Midlov P., Eilard M.S., Eriksson B., Buchebner D., Stromberg U. (2022). Targeting population groups with heavier burden of hepatocellular carcinoma incidence: a nationwide descriptive epidemiological study in Sweden. Int J Cancer.

[9] Vaz J., Hagström H., Eilard M.S., Rizell M., Strömberg U. (2025). Socioeconomic inequalities in diagnostics, care and survival outcomes for hepatocellular carcinoma in Sweden: a nationwide cohort study. Lancet Reg Health Eur.

[10] Everhov Å.H., Frisell T., Osooli M. (2025). Diagnostic accuracy in the Swedish national patient register: a review including diagnoses in the outpatient register. Eur J Epidemiol.

[11] Stromberg U., Parkes B.L., Baigi A. (2021). Small-area data on socioeconomic status and immigrant groups for evaluating equity of early cancer detection and care. Acta Oncol.

[12] Brierley J.D., Gospodarowicz M.K., Wittekind C. (2016).

[13] Valle J., Wasan H., Palmer D.H. (2010). Cisplatin plus gemcitabine versus gemcitabine for biliary tract cancer. N Engl J Med.

[14] European Commission (2013).

[15] Breslow N.E., Day N.E. (1987). Statistical methods in cancer research. Volume II--The design and analysis of cohort studies. IARC Sci Publ.

[16] Izquierdo-Sanchez L., Lamarca A., La Casta A. (2022). Cholangiocarcinoma landscape in Europe: diagnostic, prognostic and therapeutic insights from the ENSCCA registry. J Hepatol.

[17] Mihor A., Tomsic S., Zagar T., Lokar K., Zadnik V. (2020). Socioeconomic inequalities in cancer incidence in Europe: a comprehensive review of population-based epidemiological studies. Radiol Oncol.

[18] Barouki R., Samson M., Blanc E.B. (2023). The exposome and liver disease - how environmental factors affect liver health. J Hepatol.

[19] Karlsen T.H., Sheron N., Zelber-Sagi S. (2022). The EASL-lancet liver commission: protecting the next generation of Europeans against liver disease complications and premature mortality. Lancet.

[20] Konfortion J., Coupland V.H., Kocher H.M., Allum W., Grocock M.J., Jack R.H. (2014). Time and deprivation trends in incidence of primary liver cancer subtypes in England. J Eval Clin Pract.

[21] Burton A., Tataru D., Driver R.J. (2021). Primary liver cancer in the UK: incidence, incidence-based mortality, and survival by subtype, sex, and nation. JHEP Rep.

[22] Scott E.C.S., Hoskin P.J. (2024). Health inequalities in cancer care: a literature review of pathways to diagnosis in the United Kingdom. eClinicalMedicine.

[23] Hultstrand C., Brynskog E., Karlsson Rosenblad A., Sunesson A.-L., Björk-Eriksson T., Sharp L. (2025). Low levels of awareness and motivation towards cancer prevention amongst the general public in Sweden: a cross-sectional study focusing on the european code against cancer. BMC Public Health.

[24] Ludvigsson J.F., Bergman D., Lundgren C.I. (2025). The healthcare system in Sweden. Eur J Epidemiol.

[25] Nassereldine H., Compton K., Kendrick P. (2024). Burden of liver cancer mortality by county, race, and ethnicity in the USA, 2000–19: a systematic analysis of health disparities. Lancet Public Health.

[26] Rimassa L., Lamarca A., O'Kane G.M. (2025). New systemic treatment paradigms in advanced biliary tract cancer and variations in patient access across Europe. Lancet Reg Health Eur.

[27] Liu R., Zhao Y., Shi F. (2024). Cost-effectiveness analysis of immune checkpoint inhibitors as first-line therapy in advanced biliary tract cancer. Immunotherapy.

[28] Hernando-Calvo A., Nguyen P., Bedard P.L. (2024). Impact on costs and outcomes of multi-gene panel testing for advanced solid malignancies: a cost-consequence analysis using linked administrative data. eClinicalMedicine.

[29] Vogel A., Bridgewater J., Edeline J. (2023). Biliary tract cancer: ESMO clinical practice guideline for diagnosis, treatment and follow-up. Ann Oncol.

