# Supplementary material for: Socioeconomic disparities in incidence, treatment, and survival of intrahepatic cholangiocarcinoma: insights from a nationwide cohort study in Sweden
Source: Lancet Reg Health Eur. 2025 Aug 5;57:101415. doi: 10.1016/j.lanepe.2025.101415 (PMC12346144; doi:10.1016/j.lanepe.2025.101415)
Supplement: Supplementary Tables S1–S7 [file mmc1.docx]

**Socioeconomic disparities in incidence, treatment, and survival of intrahepatic cholangiocarcinoma: insights from a nationwide cohort study in Sweden**

Juan Vaz, Hannes Hagström, Per Sandström, Malin Sternby Eilard, Magnus Rizell, Ulf Strömberg

Supplementary data

| **Table of contents** | |  |
| --- | --- | --- |
| Table S1 | List of variables and definitions …………………………………………………. | 2 |
| Table S2 | Baseline characteristics of 999 patients without known risk factors diagnosed with intrahepatic cholangiocarcinoma in Sweden between 2011 and 2021……... | 4 |
| Table S3 | Likelihood of receiving surgical treatment after intrahepatic cholangiocarcinoma diagnosis …………………………………………………... | 5 |
| Table S4 | Likelihood of receiving systemic therapy after intrahepatic cholangiocarcinoma diagnosis …………….…………………………………....................................... | 6 |
| Table S5 | Likelihood of receiving palliative treatment other than systemic therapy after intrahepatic cholangiocarcinoma diagnosis ……………………………………... | 7 |
| Table S6 | Survival probabilities of patients diagnosed with intrahepatic cholangiocarcinoma in Sweden between 2011 and 2021 ……………………….. | 8 |
| Table S7 | Mortality risk after intrahepatic cholangiocarcinoma diagnosis ………………… | 10 |

| **Table S1· List of variables and definitions** | |
| --- | --- |
| **Sociodemographic characteristics** | |
|  |  |
| Sex | Male or female according to the Swedish population register |
| Age at diagnosis | Age in years |
| Country of birth | Nordic: born in Sweden, Denmark, Finland, Norway, The Faroe Island, Greenland or Iceland· Non-Nordic: born outside Nordic countries |
| Household income | Based on the distribution of household incomes for all households in Sweden·  Low (lowest quartile, <25%)  Medium (second and third quartiles, 25-75%)  High (highest quartile, >75%)  Selected for the year prior to cancer diagnosis |
|  |  |
| **Risk factors** | |
|  |  |
| Primary sclerosing cholangitis (PSC) | ICD-10: K83·0A *OR* SweLiv |
| Rare liver diseases | ICD-10: K75·4, K83·0A, K74·3, E83·0B, E88·0A, E88·0B, E83·1, K73·9, K73·2, I82·0, K76·5, K74·4, K74·5 *OR* SweLiv |
| Hepatitis B | ICD-10: B16, B17·0, B18·0, B18·1 *OR* SweLiv |
| Hepatitis C | ICD-10: B17·1, B18·2 *OR* SweLiv |
| Inflammatory bowel disease (IBD) | ICD-10: K50, K51, K52·3 *OR* SweLiv |
| PSC + IBD | ICD-10 for PSC and IBD as described above *OR* SweLiv |
| Liver cirrhosis | ICD-10: B18·0E, B18·0G, B18·1E, B18·1G, B18·2E, B18·2G, B18·8E, B18·8G, B18·9E, B18·9G, G93·4, I85·0 I85·9, I98·2, I98·3, K70·3, K71·7, K74·6, K76·6, K76·7 *AND/OR* Procedural codes: JCA20, JCA22, JCA32, JDA22 *OR* SweLiv |
| Alcohol-related liver disease | ICD-10: K70 *OR* ICD-10: E24·4, F10, G31·2, G62·1, G72·1, I42·6, K29·2, K85·2, K86·0, R78·0, T51·0, T51·9, Y57·3, Y90, X65, Z71·4, Z72·1 *OR* ATC: N07BB01 (disulfiram), N07BB03 (acamprosate), N07BB04 (naltrexone) *OR* SweLiv |
| Bile duct stones/ cyst/cholecystitis | ICD-10: K801, K802, K803, K804, K805, K808, K811, K818, K819, K915 *OR* SweLiv |
| MASLD | ICD-10: K75·8, K76·0 *OR* obesity (ICD-10: E66) *AND/OR* diabetes (as defined below) *AND* arterial hypertension *OR* hyperlipidaemia (as defined below) *OR* SweLiv *AND* No other competing liver disease· |
| None identified | No risk factor described above were identified |
|  |  |
| **Primary tumour (T)** | |
|  |  |
| TX | Primary tumour cannot be assessed |
| T0 | No evidence of primary tumour |
| Tis | Carcinoma in situ (intraductal tumour) |
| T1 | Solitary tumour without vascular invasion |
| T2 | Solitary tumour with intrahepatic vascular invasion *OR* multiple tumours, with or without vascular invasion​ |
| T3 | Tumour perforating the visceral peritoneum |
| T4 | Tumour involving local extrahepatic structures by direct invasion |
|  |  |
| **Regional lymph node (N)** | |
|  |  |
| NX | Regional lymph nodes cannot be assessed |
| N0 | No regional lymph node metastasis |
| N1 | Regional lymph node metastasis present |
|  |  |
| **Distant metastases (M)** | |
|  |  |
| MX | Distant metastases cannot be assessed |
| M0 | No evidence of metastases |
| M1 | Distant metastasis |
|  |  |
| **Stage groups (TNM)** | |
|  |  |
| Stage 0 | Tis, N0, M0 |
| Stage I | T1, N0, M0 |
| Stage II | T2, N0, M0 |
| Stage III | T3, N0, M0 OR [Any T], N1, M0 |
| Stage IV | [Any T], [Any N], M1 |
|  |  |
| **ECOG Performance status** | |
|  |  |
| 0 | Fully active, able to carry on all pre-disease performance without restriction. |
| 1 | Restricted in physically strenuous activity but ambulatory and able to perform work of a light or sedentary nature. |
| 2 | Ambulatory and capable of all self-care but unable to carry out any work activities; up and about >50% of waking hours. |
| 3 | Capable of only limited self-care; confined to bed or chair >50% of waking hours. |
| 4 | Completely disabled; cannot conduct any self-care; totally confined to bed or chair. |
|  |  |
| **Comorbidities** | |
|  |  |
| Arterial hypertension | ICD-10: I10-I15 |
| Type 2 diabetes | ICD-10: E10-E14; ATC: A10A (insulin), A10B (other glucose lowering drugs), A10X (other drugs against diabetes) *OR* SweLiv |
| Hyperlipidaemia | ICD-10: E78; ATC: C10 |
| Coronary artery disease | ICD-10: I20-I25; ATC: C01DA (organic nitrates) |
| COPD | ICD-10: J41-J44 (40 years and older) |
|  |  |
| **Treatment** | |
|  |  |
| Surgical | Resection *OR* transplantation in SweLiv *AND* ICD-10: Z94·4, T86·4 *OR* Procedural: JJC, JJB, JJA40, JJA41 |
| Systemic therapy | Systemic chemo- or immunotherapy registered in SweLiv *OR* ≥4 visits to an oncology clinic after diagnosis *AND* no surgical treatment registered |
| Other palliative | Ablation *OR* Transarterial chemoembolisation in SweLiv *OR* Procedural: JJA43, JJA44, TJJ10 *OR* radiotherapy registered in SweLiv |
| Best supportive care | No antitumour treatment reported |
|  |  |
| ATC: Anatomical Therapeutic Chemical Classification System; COPD: chronic obstructive pulmonary disease; ECOG: Eastern Cooperative Oncology Group performance status; Household income: disposable income per household per consumption unit; ICD-10: International Classification of Diseases; MASLD: metabolic dysfunction-associated steatotic liver disease; SweLiv: Swedish quality register for cancers found in the liver, gallbladder and bile ducts.  Household income refers to a person’s disposable income per consumption unit. Disposable income is the sum of all taxable and tax-free income minus taxes and negative transfers. The income includes gains/losses, i.e. the gain/loss arising from a sale (realization) of assets, for example, stocks, mutual funds or real estate. To compare disposable income and economical purchasing power between different household types, a weight system is used where consumption is related to household composition. Disposable income is divided by the weight of consumption of the household. The scale is determined by Statistics Sweden and is based on, among other things, budget calculations carried out by the Swedish Consumer Agency and the basis for assessing a basic consumption that can be calculated for different household types. | |

| **Table S2. Baseline characteristics of 999 patients without known risk factors diagnosed with intrahepatic cholangiocarcinoma in Sweden between 2011 and 2021** | | | | |
| --- | --- | --- | --- | --- |
|  | **Household income level** | | |  |
|  | **Low** | **Medium** | **High** | **Total** |
|  | 260 (26) | 488 (49) | 251 (25) | 999 (100) |
| **Male sex** | 92 (35) | 230 (47) | 120 (48) | 919 (50) |
| **Median age** | 76 (69-81) | 71 (62-77) | 65 (58-72) | 71 (62-77) |
| **Age group** |  |  |  |  |
| 18-64 | 46 (18) | 149 (31) | 120 (48) | 315 (32) |
| 65-79 | 134 (52) | 264 (54) | 115 (46) | 513 (51) |
| 80+ | 80 (30) | 75 (15) | 16 (6) | 171 (17) |
| **Country of birth** | | | | |
| Nordic | 221 (85) | 453 (93) | 238 (95) | 912 (91) |
| Non-Nordic | 39 (15) | 35 (7) | 13 (5) | 87 (9) |
| **Waiting time (in days)** |  |  |  |  |
| First suspicion to referral | 4 (0-14) | 4 (0-17) | 3 (0-14) | 4 (0-15) |
| Referral to MDT | 9 (5-18) | 8 (5-15) | 8 (6-16) | 8 (6-17) |
| MDT to surgery | 34 (27-54) | 33 (20-62) | 33 (25-53) | 33 (25-54) |
| MDT to oncology clinic | 27 (18-47) | 27 (15-48) | 20 (10-42) | 26 (14-47) |
| **Primary tumour (T)** |  |  |  |  |
| Median size (mm) | 75 (45-100) | 70 (40-100) | 75 (43-100) | 70 (40-100) |
| TX | 7 (3) | 21 (4) | 8 (3) | 36 (4) |
| T1 | 71 (27) | 128 (26) | 67 (27) | 266 (27) |
| T2 | 80 (31) | 192 (39) | 102 (40) | 374 (37) |
| T3 | 36 (14) | 76 (16) | 32 (13) | 144 (14) |
| T4 | 65 (25) | 71 (15) | 42 (17) | 179 (18) |
| **Regional lymph node (N)** |  |  |  |  |
| NX | 25 (10) | 29 (6) | 13 (5) | 67 (7) |
| N0 | 147 (56) | 281 (58) | 140 (56) | 568 (57) |
| N1 | 88 (34) | 178 (36) | 98 (39) | 364 (36) |
| **Distant metastases (M)** |  |  |  |  |
| M0 | 150 (58) | 282 (58) | 144 (57) | 576 (58) |
| M1 | 110 (42) | 206 (42) | 107 (43) | 423 (42) |
| **Staging (TNM 8^th^)** |  |  |  |  |
| Early, stage I | 45 (18) | 96 (20) | 46 (18) | 187 (19) |
| Intermediate, stages II-III | 97 (37) | 178 (36) | 94 (37) | 369 (37) |
| Late, stage IV | 110 (42) | 206 (42) | 107 (43) | 423 (42) |
| Cannot be assessed | 8 (3) | 8 (2) | 4 (2) | 20 (2) |
| **ECOG PS** | | | | |
| 0 | 38 (15) | 139 (28) | 88 (35) | 265 (27) |
| 1 | 53 (20) | 123 (25) | 63 (25) | 239 (24) |
| 2 | 114 (44) | 125 (26) | 53 (21) | 292 (29) |
| >2 or uncertain | 55 (21) | 101 (21) | 47 (19) | 203 (20) |
| **Comorbidities** | | | | |
| Arterial hypertension | 77 (30) | 114 (23) | 66 (27) | 257 (26) |
| Hyperlipidaemia | 69 (27) | 145 (30) | 66 (27) | 380 (28) |
| Coronary artery disease | 34 (13) | 47 (10) | 21 (8) | 102 (10) |
| COPD | 26 (10) | 28 (6) | 6 (2) | 60 (6) |
| Cancer other than iCCA^a^ | 19 (7) | 48 (10) | 24 (10) | 91 (9) |
| **Treatment** | | | | |
| Surgical | 29 (11) | 98 (20) | 58 (23) | 185 (18) |
| Systemic therapy | 59 (23) | 147 (30) | 89 (36) | 295 (30) |
| Other palliative treatments | 28 (11) | 73 (15) | 35 (14) | 136 (14) |
| Best supportive care | 144 (55) | 170 (35) | 69 (27) | 383 (38) |
| COPD: chronic obstructive pulmonary disease; ECOG PS: Eastern Cooperative Oncology Group performance status; iCCA: intrahepatic cholangiocarcinoma. ^a^ Diagnosed within the two years prior to the date of iCCA diagnosis. | | | | |

| **Table S3. Likelihood of receiving surgical treatment after intrahepatic cholangiocarcinoma diagnosis** | | | | |
| --- | --- | --- | --- | --- |
|  | **Univariable** | | **Multivariable** | |
|  | **OR (95% CI)** | **P-value** | **aOR (95% CI)** | **P-value** |
| **Sex** |  |  |  |  |
| Male | 1·0 (ref) | - | 1·0 (ref) | - |
| Female | 0·99 (0·79-1·24) | 0·921 | 1·28 (0·94-1·73) | 0·115 |
| **Age** | 0·97 (0·96-0·98) | <0·001 | 0·98 (0·96-0·99) | <0·001 |
| **Country of birth** |  |  |  |  |
| Nordic | 1·0 (ref) | - | 1·0 (ref) | - |
| Non-Nordic | 1·04 (0·71-1·51) | 0·825 | 0·91 (0·55-1·51) | 0·727 |
| **Household income** |  |  |  |  |
| High | 1·0 (ref) | - | 1·0 (ref) | - |
| Medium | 0·90 (0·68-1·18) | 0·455 | 1·01 (0·70-1·46) | 0·964 |
| Low | 0·60 (0·44-0·83) | 0·002 | 0·91 (0·59-1·41) | 0·682 |
| **Liver cirrhosis** | 1·38 (1·01-1·89) | 0·043 | 1·10 (0·73-1·68) | 0·637 |
| **ECOG Performance status** |  |  |  |  |
| 0 | 1·0 (ref) | - | 1·0 (ref) | - |
| 1 | 0·48 (0·36-0·63) | <0·001 | 0·64 (0·46-0·90) | 0·009 |
| ≥2 | 0·07 (0·05-0·11) | <0·001 | 0·14 (0·09-0·22) | <0·001 |
| **TNM stage** |  |  |  |  |
| I | 1·0 (ref) | - | 1·0 (ref) | - |
| II | 0·42 (0·30-0·58) | <0·001 | 0·49 (0·33-0·70) | <0·001 |
| III | 0·24 (0·18-0·33) | <0·001 | 0·27 (0·18-0·38) | <0·001 |
| CI: confidence interval; ECOG PS: Eastern Cooperative Oncology Group; OR: odds ratio. Results from univariable and multivariable logistic regression models. The multivariable model, which included all variables in this table, was statistically significant compared to the null model (Chi square (12) = 643·12, p<0·001), and correctly classified 82% of cases. | | | | |

| **Table S4. Likelihood of receiving systemic therapy after intrahepatic cholangiocarcinoma diagnosis** | | | | |
| --- | --- | --- | --- | --- |
|  | **Univariable** | | **Multivariable** | |
|  | **OR (95% CI)** | **P-value** | **aOR (95% CI)** | **P-value** |
| **Sex** |  |  |  |  |
| Male | 1·0 (ref) | - | 1·0 (ref) | - |
| Female | 1·02 (0·83-1·26) | 0·833 | 0·99 (0·78-1·26) | 0·932 |
| **Age** | 0·97 (0·96-0·98) | <0·001 | 0·97 (0·96-0·98) | <0·001 |
| **Country of birth** |  |  |  |  |
| Nordic | 1·0 (ref) | - | 1·0 (ref) | - |
| Non-Nordic | 1·26 (0·92-1·71) | 0·147 | 1·77 (1·20-2·57) | 0·004 |
| **Household income** |  |  |  |  |
| High | 1·0 (ref) | - | 1·0 (ref) | - |
| Medium | 0·91 (0·71-1·17) | 0·461 | 0·89 (0·67-1·18) | 0·413 |
| Low | 0·52 (0·39-0·71) | <0·001 | 0·54 (0·38-0·77) | 0·001 |
| **Liver cirrhosis** | 0·70 (0·50-0·97) | 0·031 | 0·71 (0·49-1·02) | 0·064 |
| **ECOG Performance status** |  |  |  |  |
| 0 | 1·0 (ref) | - | 1·0 (ref) | - |
| 1 | 1·45 (1·08-1·95) | 0·012 | 1·38 (1·01-1·88) | 0·045 |
| ≥2 | 0·45 (0·33-0·63) | <0·001 | 0·41 (0·29-0·57) | <0·001 |
| **TNM stage** |  |  |  |  |
| I | 1·0 (ref) | - | 1·0 (ref) | - |
| II | 1·58 (1·08-2·31) | 0·019 | 1·99 (1·31-3·06) | 0·001 |
| III | 2·06 (1·45-2·92) | <0·001 | 2·36 (1·59-3·52) | <0·001 |
| IV | 2·36 (1·73-3·23) | <0·001 | 3·08 (2·14-4·43) | <0·001 |
| CI: confidence interval; ECOG PS: Eastern Cooperative Oncology Group; OR: odds ratio. Results from univariable and multivariable logistic regression models. The multivariable model, which included all variables in this table, was statistically significant compared to the null model (Chi square (13) = 169·83, p<0·001), and correctly classified 74% of cases. | | | | |

| **Table S5. Likelihood of receiving palliative treatment other than systemic therapy after intrahepatic cholangiocarcinoma diagnosis** | | | | |
| --- | --- | --- | --- | --- |
|  | **Univariable** | | **Multivariable** | |
|  | **OR (95% CI)** | **P-value** | **aOR (95% CI)** | **P-value** |
| **Sex** |  |  |  |  |
| Male | 1·0 (ref) | - | 1·0 (ref) | - |
| Female | 1·11 (0·84-1·49) | 0·451 | 1·11 (0·80-1·53) | 0·533 |
| **Age** | 0·99 (0·98-1·00) | 0·057 | 0·99 (0·97-1·00) | 0·073 |
| **Country of birth** |  |  |  |  |
| Nordic | 1·0 (ref) | - | 1·0 (ref) | - |
| Non-Nordic | 0·67 (0·39-1·16) | 0·156 | 0·77 (0·43-1·38) | 0·383 |
| **Household income** |  |  |  |  |
| High | 1·0 (ref) | - | 1·0 (ref) | - |
| Medium | 1·02 (0·72-1·45) | 0·910 | 1·27 (0·84-1·91) | 0·253 |
| Low | 0·72 (0·47-1·08) | 0·114 | 0·91 (0·56-1·47) | 0·690 |
| **Liver cirrhosis** | 0·72 (0·45-1·17) | 0·185 | 0·78 (0·46-1·32) | 0·354 |
| **ECOG Performance status** |  |  |  |  |
| 0 | 1·0 (ref) | - | 1·0 (ref) | - |
| 1 | 1·56 (1·02-2·38) | 0·041 | 1·36 (0·87-2·13) | 0·182 |
| ≥2 | 1·16 (0·77-1·74) | 0·490 | 0·98 (0·62-1·55) | 0·926 |
| **TNM stage** |  |  |  |  |
| I | 1·0 (ref) | - | 1·0 (ref) | - |
| II | 1·87 (1·31-2·65) | <0·001 | 2·12 (1·04-4·35) | 0·040 |
| III | 2·50 (1·81-3·45) | <0·001 | 2·61 (1·34-5·08) | 0·005 |
| IV | 3·72 (2·79-4·98) | <0·001 | 4·62 (2·52-8·49) | <0·001 |
| CI: confidence interval; ECOG PS: Eastern Cooperative Oncology Group; OR: odds ratio. Results from univariable and multivariable logistic regression models. The multivariable model, which included all variables in this table, was statistically significant compared to the null model (Chi square (13) = 53·41, p<0·001), and correctly classified 88% of cases. | | | | |

| **Table S6. Survival probabilities of patients diagnosed with intrahepatic cholangiocarcinoma in Sweden between 2011 and 2021** | | | | | | |
| --- | --- | --- | --- | --- | --- | --- |
|  |  |  | **Survival probability (95% CI)** | | |  |
|  | **N** | **Deaths** | **1-year** | **2-year** | **5-year** | **Median survival in months (95% CI)** |
| **Surgery** |  |  |  |  |  |  |
| **Overall** | 380 | 242 | 0·86 (0·82-0·89) | 0·66 (0·60-0·70) | 0·42 (0·37-0·47) | 43 (35-50) |
| **Sex** |  |  |  |  |  |  |
| Male | 192 | 134 | 0·83 (0·77-0·88) | 0·59 (0·52-0·66) | 0·33 (0·27-0·40) | 33 (25-41) |
| Female | 188 | 108 | 0·89 (0·83-0·93) | 0·72 (0·65-0·78) | 0·51 (0·43-0·58) | 64 (45-72) |
| **Country**  **of birth** |  |  |  |  |  |  |
| Nordic | 341 | 219 | 0·87 (0·82-0·90) | 0·65 (0·60-0·70) | 0·42 (0·37-0·47) | 43 (35-51) |
| Non-Nordic | 39 | 23 | 0·82 (0·66-0·91) | 0·69 (0·52-0·81) | 0·40 (0·25-0·56) | 43 (25-) |
| **Household**  **Income** |  |  |  |  |  |  |
| High | 102 | 69 | 0·86 (0·78-0·92) | 0·62 (0·52-0·70) | 0·42 (0·32-0·51) | 41 (30-64) |
| Medium | 192 | 125 | 0·85 (0·79-0·89) | 0·65 (0·57-0·71) | 0·40 (0·33-0·47) | 41 (30-48) |
| Low | 86 | 48 | 0·88 (0·79-0·94) | 0·72 (0·61-0·80) | 0·46 (0·35-0·57) | 50 (37-81) |
| **Systemic treatment** |  |  |  |  |  |  |
| **Overall** | 503 | 483 | 0·58 (0·54-0·62) | 0·24 (0·20-0·28) | 0·04 (0·03-0·06) | 14 (13-15) |
| **Sex** |  |  |  |  |  |  |
| Male | 251 | 239 | 0·58 (0·52-0·64) | 0·26 (0·21-0·32) | 0·04 (0·02-0·07) | 13 (12-16) |
| Female | 252 | 244 | 0·58 (0·52-0·64) | 0·21 (0·17-0·27) | 0·05 (0·02-0·08) | 14 (12-15) |
| **Country**  **of birth** |  |  |  |  |  |  |
| Nordic | 438 | 422 | 0·59 (0·54-0·63) | 0·23 (0·19-0·27) | 0·04 (0·02-0·06) | 14 (12-15) |
| Non-Nordic | 65 | 61 | 0·52 (0·40-0·64) | 0·29 (0·19-0·40) | 0·05 (0·01-0·13) | 14 (9-16) |
| **Household**  **Income** |  |  |  |  |  |  |
| High | 136 | 130 | 0·59 (0·49-0·66) | 0·26 (0·19-0·33) | 0·05 (0·02-0·10) | 14 (12-17) |
| Medium | 260 | 251 | 0·61 (0·55-0·67) | 0·25 (0·20-0·30) | 0·04 (0·02-0·07) | 14 (13-16) |
| Low | 107 | 102 | 0·50 (0·41-0·59) | 0·20 (0·13-0·28) | 0·03 (0·01-0·07) | 13 (9·3-16) |
| **Other palliative treatment** |  |  |  |  |  |  |
| **Overall** | 209 | 208 | 0·12 (0·09-0·15) | 0·04 (0·02-0·06) | 0·01 (0·01-0·02) | 3·8 (3·4-4·3) |
| **Sex** |  |  |  |  |  |  |
| Male | 100 | 99 | 0·12 (0·06-0·18) | 0·04 (0·01-0·06) | 0·01 (0·01-0·05) | 3·9 (3·3-4·7) |
| Female | 109 | 109 | 0·06 (0·03-0·12) | 0·01 (0·01-0·05) | - | 3·7 (3·3-4·6) |
| **Country**  **of birth** |  |  |  |  |  |  |
| Nordic | 194 | 193 | 0·09 (0·39-0·44) | 0·04 (0·02-0·06) | 0·01 (0·01-0·02) | 3·8 (3·4-4·4) |
| Non-Nordic | 15 | 15 | 0·07 (0·01-0·26) | 0·00 | 0·00 | 4·1 (2·7-6·8) |
| **Household**  **Income** |  |  |  |  |  |  |
| High | 52 | 51 | 0·17 (0·09-0·29) | 0·06 (0·02-0·14) | 0·02 (0·01-0·09) | 3·8 (3·2-5·1) |
| Medium | 108 | 108 | 0·08 (0·04-0·14) | 0·00 | 0·00 | 4·0 (3·5-4·7) |
| Low | 49 | 49 | 0·00 | 0·00 | 0·00 | 3·6 (3·2-4·3) |
| **BSC** |  |  |  |  |  |  |
| **Overall** | 735 | 722 | 0·16 (0·13-0·19) | 0·07 (0·05-0·08) | 0·02 (0·01-0·03) | 2·9 (2·7-3·5) |
| **Sex** |  |  |  |  |  |  |
| Male | 376 | 369 | 0·14 (0·10-0·17) | 0·06 (0·04-0·09) | 0·02 (0·01-0·04) | 2·9 (2·5-3·3) |
| Female | 359 | 354 | 0·19 (0·15-0·23) | 0·08 (0·05-0·11) | 0·02 (0·01-0·04) | 3·3 (2·7-3·8) |
| **Country**  **of birth** |  |  |  |  |  |  |
| Nordic | 672 | 661 | 0·16 (0·14-0·19) | 0·06 (0·05-0·08) | 0·02 (0·01-0·03) | 2·9 (2·7-3·4) |
| Non-Nordic | 63 | 62 | 0·14 (0·07-0·24) | 0·09 (0·04-0·18) | 0·03 (0·01-0·09) | 3·8 (2·5-5·1) |
| **Household**  **Income** |  |  |  |  |  |  |
| High | 134 | 130 | 0·19 (0·13-0·26) | 0·07 (0·04-0·13) | 0·03 (0·01-0·08) | 10 (8·4-13) |
| Medium | 305 | 302 | 0·13 (0·09-0·17) | 0·04 (0·02-0·07) | 0·01 (0·01-0·02) | 8·9 (8·1-10) |
| Low | 296 | 291 | 0·18 (0·14-0·23) | 0·09 (0·06-0·12) | 0·02 (0·01-0·04) | 6·3 (5·3-7·8) |
| BSC: best supportive care; CI: confidence interval. | | | | | | |

| **Table S7. Mortality risk after intrahepatic cholangiocarcinoma diagnosis** | | | | |
| --- | --- | --- | --- | --- |
|  | **Univariable** | | **Multivariable** | |
|  | **HR (95% CI)** | **P-value** | **aHR (95% CI)** | **P-value** |
| **Surgical treatment** | | | | |
| **Sex** |  |  |  |  |
| Male | 1·0 (ref) | - | 1·0 (ref) | - |
| Female | 0·66 (0·51-0·84) | 0·001 | 0·65 (0·50-0·84) | 0·001 |
| **Age** | 1·00 (0·99-1·01) | 0·282 | 1·00 (0·99-1·02) | 0·132 |
| **Country of birth** |  |  |  |  |
| Nordic | 1·0 (ref) | - | 1·0 (ref) | - |
| Non-Nordic | 0·96 (0·63-1·48) | 0·871 | 1·03 (0·66-1·60) | 0·891 |
| **Household income** |  |  |  |  |
| High | 1·0 (ref) | - | 1·0 (ref) | - |
| Medium | 0·97 (0·72-1·30) | 0·840 | 0·98 (0·73-1·32) | 0·913 |
| Low | 0·79 (0·55-1·14) | 0·211 | 0·79 (0·54-1·17) | 0·245 |
| **Systemic therapy** | | | | |
| **Sex** |  |  |  |  |
| Male | 1·0 (ref) | - | 1·0 (ref) | - |
| Female | 1·05 (0·88-1·26) | 0·594 | 1·02 (0·85-1·23) | 0·824 |
| **Age** | 1·00 (0·99-1·01) | 0·981 | 1·00 (0·99-1·01) | 0·629 |
| **Country of birth** |  |  |  |  |
| Nordic | 1·0 (ref) | - | 1·0 (ref) | - |
| Non-Nordic | 0·99 (0·76-1·30) | 0·959 | 0·93 (0·70-1·23) | 0·599 |
| **Household income** |  |  |  |  |
| High | 1·0 (ref) | - | 1·0 (ref) | - |
| Medium | 1·06 (0·86-1·32) | 0·575 | 1·07 (0·87-1·33) | 0·517 |
| Low | 1·38 (1·08-1·67) | 0·019 | 1·34 (1·09-1·65) | 0·011 |
| **Other palliative treatments** | | | | |
| **Sex** |  |  |  |  |
| Male | 1·0 (ref) | - | 1·0 (ref) | - |
| Female | 1·05 (0·80-1·39) | 0·688 | 1·03 (0·78-1·37) | 0·815 |
| **Age** | 1·01 (0·99-1·02) | 0·392 | 1·00 (0·99-1·02) | 0·718 |
| **Country of birth** |  |  |  |  |
| Nordic | 1·0 (ref) | - | 1·0 (ref) | - |
| Non-Nordic | 0·94 (0·56-1·59) | 0·822 | 0·88 (0·50-1·54) | 0·660 |
| **Household income** |  |  |  |  |
| High | 1·0 (ref) | - | 1·0 (ref) | - |
| Medium | 1·34 (0·95-1·89) | 0·096 | 1·36 (0·96-1·93) | 0·083 |
| Low | 1·58 (1·04-2·38) | 0·029 | 1·56 (1·02-2·38) | 0·040 |
| **Best supportive care** | | | | |
| **Sex** |  |  |  |  |
| Male | 1·0 (ref) | - | 1·0 (ref) | - |
| Female | 0·90 (0·78-1·05) | 0·177 | 0·92 (0·79-1·06) | 0·221 |
| **Age** | 1·00 (0·99-1·01) | 0·646 | 1·00 (0·99-1·01) | 0·508 |
| **Country of birth** |  |  |  |  |
| Nordic | 1·0 (ref) | - | 1·0 (ref) | - |
| Non-Nordic | 0·88 (0·68-1·14) | 0·341 | 0·91 (0·70-1·19) | 0·502 |
| **Household income** |  |  |  |  |
| High | 1·0 (ref) | - | 1·0 (ref) | - |
| Medium | 1·09 (0·89-1·34) | 0·394 | 1·08 (0·87-1·33) | 0·481 |
| Low | 0·94 (0·76-1·15) | 0·515 | 0·93 (0·75-1·16) | 0·526 |
| CI: confidence interval; HR: hazard ratio. Results from univariable and multivariable Cox regression models. The multivariable models included all variables in this table. | | | | |
